# The specificity of the auditory P300 responses and its association with clinical outcomes in youth with psychosis risk syndrome

**DOI:** 10.1016/j.ijchp.2024.100437

**Published:** 2024-01-11

**Authors:** Yongqing Hou, Guiping Qiu, Haishuo Xia, Tianbao He, Xiaoxian Liu, Antao Chen

**Affiliations:** a Key Laboratory of Cognition and Personality of Ministry of Education, Faculty of Psychology, Southwest University, Chongqing, China; b Mental Health Center of Guangyuan, Sichuan, China; c College of Teacher Education, Ningxia University, Yinchuan, China; d Faculty of Education, Henan Normal University, Xinxiang, China; e School of Psychology, Research Center for Exercise and Brain Science, Shanghai University of Sport, Shanghai, China

**Keywords:** Psychosis risk syndrome (PRS), Auditory attention, P300, 40-Hz stimulation, Oddball paradigm

## Abstract

**Background:**

Schizophrenia often occurs in youth, and psychosis risk syndrome (PRS) occurs before the onset of psychosis. Assessing the neuropsychological abnormalities of PRS individuals can help in early identification and active intervention of mental illness. Auditory P300 amplitude defect is an important manifestation of attention processing abnormality in PRS, but it is still unclear whether there are abnormalities in the attention processing of rhythmic compound tone stimuli in PRS individuals, and whether the P300 amplitude induced by these stimuli is specific to PRS individuals and related to their clinical outcomes.

**Methods:**

In total, 226 participants, including 122 patients with PRS, 51 patients with emotional disorders (ED), and 53 healthy controls (HC) were assessed. Baseline electroencephalography was recorded during the compound tone oddball task. The event-related potentials (ERPs) induced by rhythmic compound tone stimuli of two frequencies (20-Hz, 40-Hz) were measured. Almost all patients with PRS were followed up for 12 months and reclassified into four groups: PRS-conversion, PRS-symptomatic, PRS-emotional disorder, and PRS-complete remission. The differences in baseline ERPs were compared among the clinical outcome groups.

**Results:**

Regardless of the stimulation frequency, the average P300 amplitude were significantly higher in patients with PRS than in those with ED (*p* = 0.003, *d* = 0.48) and in HC (*p* = 0.002, *d* = 0.44) group. The average P300 amplitude of PRS-conversion group was significantly higher than that of the PRS-complete remission (*p* = 0.016, *d* = 0.72) and HC group (*p* = 0.001, *d* = 0.76), and the average P300 amplitude of PRS-symptomatic group was significantly higher than that of the HC group (*p* = 0.006, *d* = 0.48). Regardless of the groups (PRS, ED, HC) or the PRS clinical outcome groups, the average P300 amplitude induced by 20-Hz tone stimulation was significantly higher than that induced by 40-Hz stimulation (*p*s < 0.001, Ƞ^2^ = 0.074–0.082). The average reaction times of PRS was significantly faster than that of ED (*p* = 0.01, *d* = 0.38), and the average reaction times of the participants to 20-Hz target stimulation was significantly faster than that to 40-Hz target stimulation (*p* < 0.001, *d* = 0.21).

**Conclusion:**

The auditory P300 amplitude induced by rhythmic compound tone stimuli is a specific electrophysiological manifestation of PRS, and the auditory P300 amplitude induced by compound tone stimuli shows promise as a putative prognostic biomarker for PRS clinical outcomes, including conversion to psychosis and clinical complete remission.

## Introduction

Most psychiatric disorders, especially schizophrenia, usually have a prodrome before reaching the clear diagnostic standard (Bosnjak Kuharic, Kekin, Hew, Rojnic Kuzman & Puljak, 2019; Worthington et al., 2021), consisting of attenuated psychotic symptoms and/or a decline in premorbid functioning (Catalan et al., 2021; Fusar-Poli et al., 2020). This prodrome is usually called the clinical high-risk, ultra-high risk, or psychosis risk syndrome (PRS) (Bang et al., 2015; Miller et al., 2003; Yung et al., 2005). PRS may be an intermediate state between psychosis and health, and approximately 80 % ∼ 90 % of schizophrenic patients have a prodrome period of 1 ∼ 5 years before reaching the disease diagnostic criteria (De Herdt et al., 2013; Perkins et al., 2020). Moreover, youth with PRS are at high risk for psychosis (Addington et al., 2011). However, approximately only 15 % ∼ 36 % of PRS individuals will become psychiatric patients within two years (Fusar-Poli et al., 2012; Nelson et al., 2013), which limits the focalization and justification of early intervention on PRS, particularly regarding early treatment with antipsychotic medications. Therefore, in recent years, researchers have begun to consider improving the PRS diagnostic criteria through biomarkers to lay the foundation for proactive early intervention among individuals at high risk of mental illness (Bodatsch, Brockhaus-Dumke, Klosterkötter & Ruhrmann, 2015; Kraguljac et al., 2021). In addition, due to the PRS is associated with a variety of clinical outcomes ranging from psychosis to remission from the risk state (Nelson et al., 2013), identifying biomarkers related to different clinical outcomes of PRS can improve the clinical prognosis accuracy of PRS, which may promote the advancement of staging treatment algorithms and make the prognosis and treatment plans for PRS more refined and precise. Moreover, clarifying such biomarkers may help elucidate the pathophysiological processes involved in the occurrence and development of mental illness (especially schizophrenia), thereby guiding the development of new targeted interventions.

Abnormal attention processing is a key factor for positive symptoms of psychosis and the main reason for cognitive impairment (Carbon & Correll, 2014; Orellana & Slachevsky, 2013). Attention abnormalities may have a significant impact on the occurrence, development, and outcome of PRS (H. K. Hamilton et al., 2019). Numerous studies have found that patients with schizophrenia exhibit significant auditory attention processing abnormalities, showed trait like reduction in auditory P300 event-related potentials (ERP) amplitude (Bramon et al., 2005; H. K. Hamilton et al., 2019; D. H. Mathalon, Ford & Pfefferbaum, 2000; Mohn & Torgalsbøen, 2018; Wood et al., 2007). The auditory P300 amplitude is an important candidate electrophysiological biomarker for mental disorders (Bramon et al., 2005; H. K. Hamilton et al., 2019). Typically elicited during an oddball target detection task by infrequently presented salient stimuli interspersed among frequent standard stimuli, the P300 response is a positive voltage deflection in the stimulus-locked ERP occurring 300 milliseconds after the stimulus, and typically reaching its maximum in the frontoparietal or parietooccipital scalp (Huang, Chen & Zhang, 2015; Polich, 2007; van Dinteren, Arns, Jongsma & Kessels, 2014). The P300 amplitude is thought to reflect attention/alertness, allocation of attention resources, attention transfer, working memory refresh, and other cognitive psychological processes (Bachiller et al., 2015; Huang et al., 2015; Luck, Woodman & Vogel, 2000), and P300 latency reflects the speed and efficiency of information processing (Leuthold & Sommer, 1998; Verleger, 1997). In individuals with PRS, attention abnormality is lower than that in patients with schizophrenia, but shows a certain degree of neurophysiological deficits (H. K. Hamilton et al., 2019; Tor et al., 2018, 2020). Previous studies have reported that a P300 amplitude defect is an important manifestation of abnormal attention in individuals with PRS and plays an important role in predicting the conversion of PRS to psychosis (H. K. Hamilton et al., 2019, 2019).

However, previous studies using the auditory oddball paradigm have mostly used monotonic pure tone stimuli as experimental materials (H. K. Hamilton et al., 2019; Kayser et al., 2014) and rarely used compound tone stimuli (a continuous sound sequence composed of multiple monotonic pure tones, such as click tones), therefore, it is not clear whether PRS individuals have abnormal attentional processing to compound tone stimuli as well as pure tone stimuli. Compared with pure tone stimulation, compound tone stimulation has unique physical characteristics, contains more abundant auditory information, and brings more unique auditory experience to individuals (Voicikas, Niciute, Ruksenas & Griskova-Bulanova, 2016). Moreover, the cerebral cortex exhibits different characteristics when processing pure and compound tones (Vihla & Salmelin, 2003). Compared to pure tone stimuli, processing compound tone stimuli requires more cognitive resources to invest (Blanchet, 2015; Christoff, 1999; Wickens, 1980), the level of cognitive resources invested by individuals has an important effect on their neurophysiological responses (Lenartowicz, Simpson, Haber & Cohen, 2014). For the cognitive processing performance of psychosis and PRS individuals, individuals with psychiatric symptoms typically exhibit slower processing of information with less demand for cognitive resources and more sensitive processing of information with more demand for cognitive resources (Aase et al., 2021; Addington et al., 2019; Vaquerizo-Serrano, Salazar de Pablo, Singh & Santosh, 2022). Therefore, attention processing of compound tone stimuli may reflect new features of auditory attention abnormalities in PRS. However, there is still a lack of research on this issue at present.

In addition, studies focusing on the auditory steady-state response (ASSR) caused by rhythmic compound tone stimuli have found that compared to other frequency tone stimuli, rhythmic tone stimuli at 40-Hz can induce stronger gamma band neural oscillatory responses in individuals (Cortes-Briones, 2021; Pastor et al., 2002; Voicikas et al., 2016). Related studies have found significant ASSR deficits in patients with schizophrenia, revealing possible abnormal synchronous activity of auditory cortical neurons in patients (Grent-‘t-Jong, Brickwedde, Metzner & Uhlhaas, 2023; O'Donnell et al., 2013; Thuné, Recasens & Uhlhaas, 2016). Some studies have also found that first-episode schizophrenia patients, first-episode emotional disorders patients, and PRS individuals all have ASSR deficits (Grent-’t-Jong et al., 2021; Spencer, Salisbury, Shenton & McCarley, 2008; Tada et al., 2016), and ASSR induced by 40-Hz rhythmic tone stimulation can more effectively predict the clinical outcomes of PRS (Cortes-Briones, 2021; Grent-’t-Jong et al., 2021). Therefore, rhythmic compound tone stimulation at 40-Hz may have a unique role in assessing the neurophysiological responses related to auditory attention processing in patients with schizophrenia and PRS. However, there are currently few studies analyzing the auditory attention processing characteristics of individuals with PRS to rhythmic compound tone stimuli of different frequencies, and there is also a lack of analysis on the association between the P300 response induced by rhythmic compound tone stimuli of different frequencies and the clinical outcomes of PRS.

Importantly, previous studies only compared the attention processing characteristics of PRS and healthy controls (HC) individuals, but not among those with other mental disorders. In this case, the specificity of PRS attention processing and the corresponding neurophysiological mechanism is lacking (Beck et al., 2019; Bora et al., 2014). Accordingly, there is no evidence to show which ERP response defects are unique to PRS individuals. In addition to having positive symptoms, PRS individuals often exhibit a certain degree of negative symptoms (such as social isolation and lack of will), disintegrating symptoms (such as strange behavior and difficulty concentrating), and general symptoms (such as sleep disorders and emotional abnormalities), which usually appear before or accompanied by positive symptoms (McGlashan et al., 2006). These non-positive symptoms can reflect the severity of psychological abnormalities in PRS individuals (Gupta, Cowan, Strauss, Walker & Mittal, 2021), and also have a certain impact on the clinical outcomes of PRS individuals (Nelson et al., 2013; Zhang et al., 2021). The majority of PRS individuals have non-positive symptoms mainly characterized by anxiety and depression emotional symptoms, and the comorbidity of anxiety and depressive disorders with PRS is very common (Fusar-Poli, Nelson, Valmaggia, Yung & McGuire, 2014). Compared with PRS individuals, those with emotional disorders (ED) do not have the positive symptoms of PRS individuals; instead, they experience negative, disintegrating, and general symptoms such as emotional abnormality and abulia (Critchley, 2003; Rolls, 2021). Thus, including the ED group in the research design and comparing the P300 responses between the PRS group and ED group may help to identify the relationship between PRS positive symptoms and attention processing and more clearly explore the specificity of PRS attention processing and its neurophysiological mechanism.

Accordingly, the present study used a new auditory oddball task using rhythmic compound tone stimuli as experimental material to evaluate the P300 response of PRS, ED, and HC individuals. To explore the specificity of PRS attention processing and its neurophysiological mechanism, the differences in P300 responses were compared among the three groups of participants. By comparing the P300 responses induced by rhythmic compound tone stimuli at two frequencies (20-Hz and 40-Hz), the auditory attention processing characteristics of participants to compound tone stimuli at different frequencies were analyzed. Furthermore, after a 12-month follow-up of PRS individuals, by analyzing the association between P300 amplitude at the baseline level and the clinical outcomes of PRS, the electrophysiological indicators associated with the clinical outcomes of PRS were confirmed.

Due to the fact that processing compound tone stimuli requires more cognitive resources compared to pure tone stimuli (Blanchet, 2015; Christoff, 1999; Wickens, 1980), and individuals with psychiatric symptoms typically exhibit slower processing of information with less cognitive resource requirements and more sensitive processing of information with more cognitive resource requirements (Aase et al., 2021; Addington et al., 2019; Vaquerizo-Serrano et al., 2022). Therefore, we hypothesized that compared to HC and ED individuals, the P300 amplitude induced by rhythmic compound tone stimuli was greater in PRS individuals. Because in compound sound stimulation, high-frequency target sound is more difficult to distinguish from standard sound with the same frequency than low-frequency target sound (Tada et al., 2016). Therefore, we hypothesized that compared to the P300 amplitude induced by rhythmic compound tone stimuli at 20-Hz, the P300 amplitude induced by compound tone stimuli at 40-Hz was smaller. In addition, previous studies have found that compared to other frequency sound stimuli, rhythmic sound stimuli at 40-Hz can induce stronger gamma band neural oscillation responses in individuals (O'Donnell et al., 2013; Pastor et al., 2002; Voicikas et al., 2016), and Gamma neural oscillations are closely related to attentional processing (Fell, Fernández, Klaver, Elger & Fries, 2003; Parciauskaite, Bjekic & Griskova-Bulanova, 2021). Furthermore, ASSR induced by 40-Hz rhythmic tone stimuli can more effectively predict the clinical outcomes of PRS (Cortes-Briones, 2021; Grent-’t-Jong et al., 2021). Therefore, we hypothesized that after 12 months of follow-up, compared to PSR individuals who did not convert to psychosis, PRS individuals who converted to psychosis had a greater baseline P300 amplitude. Moreover, compared to PRS individuals who converted to psychosis, individuals who were completely relieved from PRS status exhibited smaller baseline P300 amplitudes.

## Materials and methods

### Participants

In total, 226 participants were selected from the psychological survey results of 8763 freshmen in three Chinese universities. Participants with PRS met the criteria for Psychosis-Risk Syndromes based on the Chinese version of the Structured Interview for Psychosis-risk Syndromes (SIPS), and symptoms were rated using the Scale of Psychosis-risk Symptoms (SOPS) (Zheng et al., 2012). The Chinese version of the Mini-International Neuropsychiatric Interview (MINI) (Si et al., 2009) was used to exclude patients with other mental disorders. For ED, the enrolled participants had to meet the diagnosis of current depressive episodes, have symptoms of generalized anxiety disorder, and meet the following requirements: Patient Health Questionnaire-9 (Kroenke, Spitzer & Williams, 2001) total score ≥10; Hamilton Depression Rating Scale (Williams & Janet, 1988) score ≥17; Self-Rating Depression Scale (Zung, 1965) standard score ≥63; and/or the Generalized Anxiety Disorder Scale-7 (Spitzer, Kroenke, Williams & Löwe, 2006) total score ≥10, Hamilton Anxiety Rating Scale (Snaith, Baugh, Clayden, Husain & Sipple, 1982) score ≥14, and Self-Rating Anxiety Scale (Zung, 1971) standard score ≥60. Moreover, individuals with ED did not have any type of PRS assessed by SIPS and had no other mental disorders assessed by MINI. HCs did not have a first-degree relative with a psychotic disorder and were not currently receiving antipsychotic medications. All participants who 1) had nervous system diseases; 2) had a history of traumatic brain injury; 3) had taken psychotropic drugs in the past year; 4) had received electroconvulsive therapy; 5) were pregnant or lactating; 6) had a history of alcohol abuse or dependence; or 7) had a history of using or relying on heroin, morphine, or similar substances were excluded. Before the study, a 1000 Hz sound of 30 dB was successfully detected by all participants. Specific diagnostic information for participants are presented in the Supplementary Materials. Written informed consent was obtained from all participants. The study was approved by the ethical review of the Ethics Committee of the Faculty of Psychology, Southwest University (IRB No. H20061) and the Medical Ethics Committee of Guangyuan Mental Health Center (IRB No. GJW20210303009). Data were collected from November 15, 2020, to May 30, 2022. This study has been preregistered in OSF (doi:10.17605/OSF.IO/6DT24).

Twelve months after the initial assessment, all participants with PRS were again assessed using SIPS. In total, 121 individuals with PRS participated in the assessment (one person was lost to follow-up). After the assessment, 24 (19.84 %) individuals were classified as psychotic and met the criteria for psychiatric symptoms (PRS-conversion group), 56 (46.28 %) individuals still met the criteria for PRS (PRS-symptomatic group) in the past 4 weeks, and 41 (33.88 %) individuals were relieved of symptoms and no longer met the PRS criteria (PRS-remission group). In the PRS-remission group, according to the residual condition of symptoms, 19 (15.70 %) patients had mild remission of ED (PRS-emotional disorder group) and 22 (18.18 %) had complete remission (PRS-complete remission group). The PRS-emotional disorder group was relieved of positive symptoms, but still showed obvious negative, general, and disintegrating symptoms, and the PRS-complete remission group was completely relieved of PRS without any obvious symptoms.

### Stimulation and procedures

**Fig. 1:**
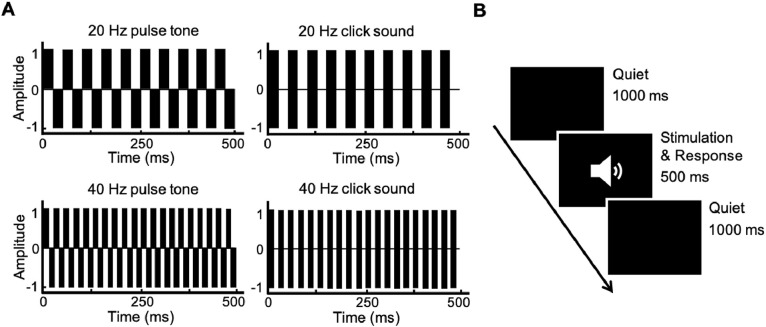
The task process and materials. (A) The schematic representation of stimuli used in the study. (B) Task process.

Two types of rhythmic compound tones (pulse train, click train) with two frequencies (20-Hz, 40-Hz) were used as experimental stimuli for auditory Oddball tasks (Korczak, Smart, Delgado, Strobel & Bradford, 2012). The schematic diagram of the stimuli is shown in Fig. 1a. The testing program was designed using E-prime 2.0 software. The formal testing was divided into two blocks based on tone frequency, and the presentation order of the blocks was random among the participants. In a block, pulse trains are the standard stimulus, and click trains of the same frequency are the target stimulus (under the same tone frequency, click train have higher loudness than pulse tone, and participants experience more significant rhythmic fluctuations in click train). Each block has 130 trials, including 30 trials of target stimulation. Each trial consists of a 500 ms tone stimulus (with a volume of 60 dB) and a 1000 ms quiet state, the trial of the target stimulus appears randomly. The presentation process of the stimuli is shown in Fig. 1b. The sound stimuli were presented through air-conducting earphones. Participants were asked to press the space bar quickly and accurately upon hearing the target stimulus. Throughout the entire test, participants completed it in a quiet, comfortable, and electromagnetically shielded room.

### EEG acquisition and processing

Participants were required to refrain from smoking, drinking, or taking sedative and hypnotic drugs for 12 h before data collection. Electroencephalogram (EEG) data were collected using a standard silver/silver chloride 64-channel electrode cap (Neuroscan). Horizontal electro-oculogram (HEOG) was recorded for both eyes, and vertical electro-oculogram (VEOG) was recorded above and below the left eye; the reference electrode was CZ. The signal acquisition impedance was adjusted to below 5 kΩ, the band-pass filter was 0.01–100 Hz, and the sampling rate was 1000 Hz/channel.

The EEGLAB 14.1.1b toolbox (http://sccn.ucsd.edu/eeglab/) based on MATLAB software was used to process the collected EEG data offline (Delorme & Makeig, 2004). EEG data were first filtered by band-pass filtering at 0.1–49 Hz and then re-referenced according to the whole brain average reference. The automatic artifact removal 1.3 (http://www.cs.tut.fi/~gomezher/index.htm) plug-in was used to remove artifacts such as electrooculogram and electromyogram (Gomez-Herrero, 2007). EEG data were segmented according to the stimulus events. The auditory stimulus onset was at 0 ms; 200 ms was reserved before stimulus onset, and 800 ms was reserved after stimulus presentation. The ERPs were baseline corrected (−200–0 ms). Trials with EEG amplitudes exceeding 100 µV were removed (standard stimulus trials removed: 20-Hz has an average of 9.83, and 40-Hz has an average of 11.39; target stimulus trials removed: 20-Hz has an average of 3.01, and 40-Hz has an average of 3.52), and all remaining trials were included in the subsequent analysis.

The EEGLAB plug-in ERPLAB toolbox (http://erpinfo.org/erplab) was used to calculate the average ERPs induced by the target and standard stimuli, respectively (Javier & Luck, 2014). To obtain a clear ERP index and generate the P300, the standard stimulation amplitude was subtracted from the target stimulation amplitude at 300–400 ms after stimulation. The values were taken from four electrodes (P1, PZ, P2, and POZ, where P300 are the largest) in the parietal–occipital region and the grand average ERP was defined as the P300 amplitude (average within the time window and electrodes). The grand average time corresponding to the maximum P300 amplitude at each electrode was the P300 latency.

### Statistical analysis

We used one-way ANOVA to analyze the questionnaire data according to the groups (PRS, ED, HC). A 3 (Group: PRS, ED, HC) × 2 (Stimulus frequency: 20-Hz, 40-Hz) two-factor repeated measurement analysis of variance (ANOVA) was used to analyze the reaction times (RTs) and accuracies of target stimuli detection and the P300 amplitude and latency. In addition, we re-sorted PRS participants based on clinical outcomes after one year of follow-up and divided them into four clinical outcome groups: PRS-conversion, PRS-symptomatic, PRS-emotion disorder, and PRS-complete remission. Using the clinical outcome group of patients with PRS after follow-up as the among-subjects variable and stimulus frequency as the within-subjects variable, repeated measurement ANOVA was performed for the P300 amplitude and latency. We used the least significant difference (LSD) method to control possible multiple comparisons in the post-hoc test. However, for across ANOVA tests, we did not control for multiple comparisons, therefore only nominal p-values are reported. Pearson correlation (2-tailed) was used to analyze the correlation between the severity of PRS symptoms and P300 response. In all analyses, *p* <  0.05 was statistically significant. SPSS 25.0 (IBM Corp., Armonk, NY) was used for statistical analyses.

## Results

### Sample characteristics

The demographic characteristics of all participants and clinical outcome groups of PRS after follow-up are presented in Table 1 and Table A.1 in the Supplementary Materials respectively. Age, sex, home location, father's education, and mother's education did not differ significantly by group or clinical outcome group. The statistical results of anxiety, depression, and SOPS scores for PRS, ED, and HC individuals are shown in Table A.2 in the Supplementary Materials.

**Table 1:** Demographic statistics of participants.

Characteristic	PRS (*n* = 122)	ED (*n* = 51)	HC (*n* = 53)	*P*-value
Age in years, mean (*SD*)	18.44(0.99)	18.65(0.80)	18.64(0.90)	0.27
Sex assigned at birth, no. ( %)				0.42
Male	41(33.6 %)	17(33.3 %)	23(43.4 %)	
Female	81(66.4 %)	34(66.7 %)	30(56.6 %)	
Home location, no. ( %)				0.43
City	46(37.7 %)	23(45.1 %)	27(50.9 %)	
Urban rural fringe	23(18.9 %)	6(11.8 %)	6(11.3 %)	
Countryside	53(43.4 %)	22(43.1 %)	20(37.7 %)	
Father's education, no. ( %)				0.14
Junior high school and below	66(54.1 %)	23(45.1 %)	25(47.2 %)	
High school and technical secondary school	28(23.0 %)	20(39.2 %)	11(20.8 %)	
Junior college	14(11.5 %)	1(2.0 %)	8(15.1 %)	
Undergraduate	13(10.7 %)	6(11.8 %)	9(17.0 %)	
Graduate and above	1(0.8 %)	1(2.0 %)	0(0.0 %)	
Mother's education, no. ( %)				0.26
Junior high school and below	71(58.2 %)	33(64.7 %)	26(49.1 %)	
High school and technical secondary school	32(26.2 %)	13(25.5 %)	13(24.5 %)	
Junior college	11(9.0 %)	1(2.0 %)	10(18.9 %)	
Undergraduate	7(5.7 %)	4(7.8 %)	4(7.5 %)	
Graduate and above	1(0.8 %)	0(0.0 %)	0(0.0 %)	

Note: Data are number (percentage) or mean (SD), when appropriate. P-values by ANOVA linear term or Pearson Chi square tests (for linear association). Significant at *p* < 0.05. PRS = psychosis risk syndrome; ED = emotional disorder; HC = healthy control.

### P300 amplitude and latency

**Fig. 2:**
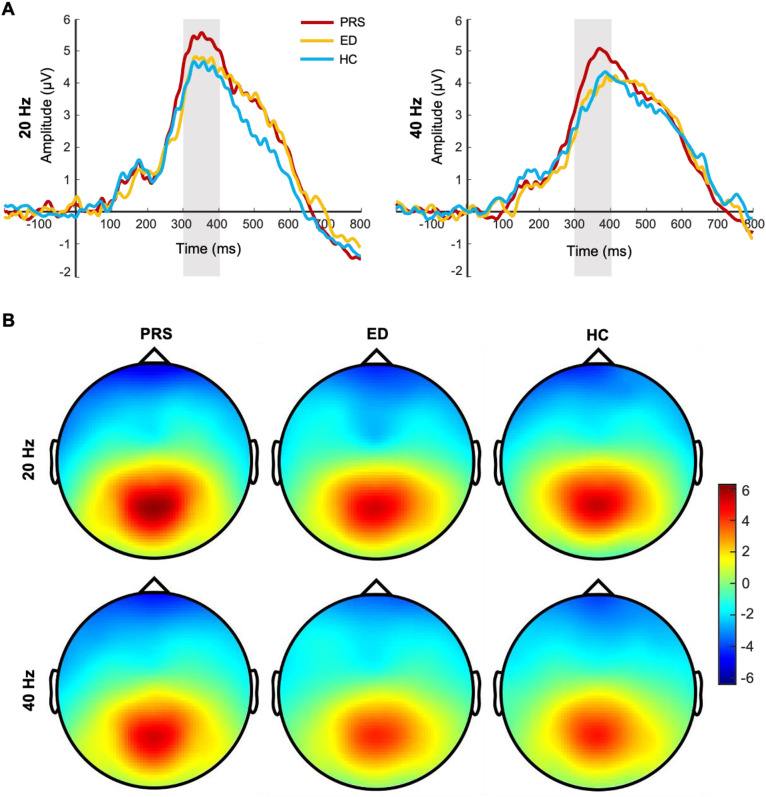
The difference waveforms and topographic maps of each group for different stimulus frequencies. (A) The P300 amplitudes of PRS, ED, and HC for different stimulus frequencies. The amplitudes are the average of P1, PZ, P2, and POZ electrodes. (B) The topographic maps of PRS, ED, and HC for different stimulus frequencies. The time window of topographic maps is 300–400 ms.

For the P300 amplitude, the main effect of the group (PRS, ED, HC) was significant (*F*(2, 225) = 7.16, *p* = 0.001, Ƞ^2^ =  0.06). Post-tests showed that the P300 amplitude was significantly greater in patients with PRS than in those with ED (*p* *=* 0.003, *d* *=* 0.48) and HC (*p* *=* 0.002, *d* = 0.44). There was no significant difference in the P300 amplitude between patients with ED and HC (*p* *=* 0.98, *d* = 0.01). The main effect of stimulus frequency was significant (*F*(1, 225) = 19.79, *p*  *<*  0.001, Ƞ^2^ = 0.08); the P300 amplitude induced by 20-Hz sound stimulation was significantly greater than that induced by 40-Hz sound stimulation. The interaction between group and stimulus frequency was not significant (*F*(2, 224) = 0.65, *p* *=* 0.52, Ƞ^2^ = 0.006). In terms of latency, the main effect of the group was marginal significant (*F*(2, 225) = 3.00, *p* = 0.052, Ƞ^2^ = 0.03). Post-tests showed that the latency of PRS was smaller than that of HC (*p* *=* 0.03, *d* *=* 0.27); there was no significant difference in the latency between PRS and ED (*p* *=* 0.08, *d* *=* 0.22), and ED and HC (*p* *=* 0.78, *d* = 0.04). The main effect of stimulus frequency was significant (*F*(1, 225) = 20.45, *p*  *<*  0.001, Ƞ^2^ = 0.08); the P300 latency induced by 20-Hz sound stimulation was significantly smaller than that induced by 40-Hz sound stimulation. The interaction between group and stimulus frequency was not significant (*F*(2, 224) = 0.48, *p* *=* 0.62, Ƞ^2^ = 0.004). The difference waveforms and topographic maps of each group with different stimulus frequencies are shown in Fig. 2.

**Fig. 3:**
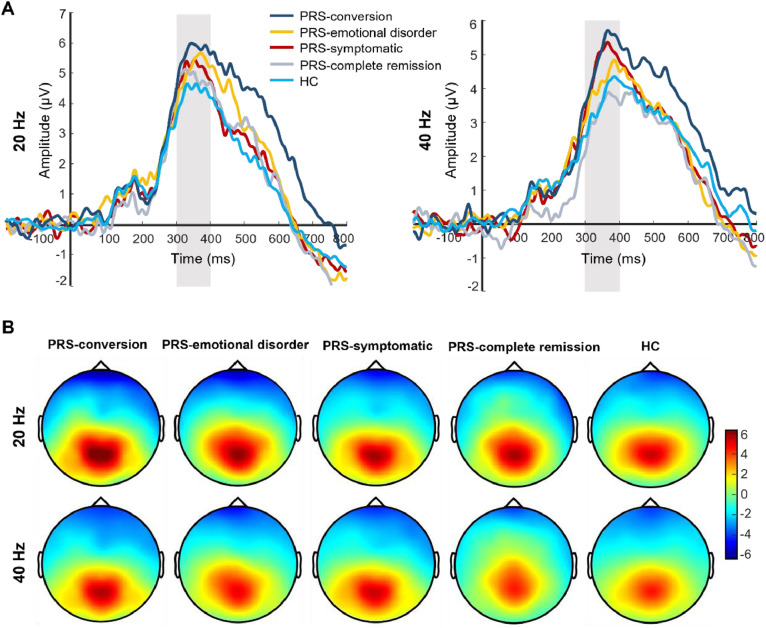
The difference waveforms and topographic maps of each group for different stimulus frequencies after follow-up. (A) The P300 amplitudes of each group for different stimulus frequencies after follow-up. The amplitudes are the average of P1, PZ, P2, and POZ electrodes. (B) The topographic maps of each group for different stimulus frequencies after follow-up. The time window of topographic maps is 300–400 ms.

After follow-up, the main effect of the clinical outcome group was significant in P300 amplitude (*F*(4, 173) = 3.81, *p* *=* 0.005, Ƞ^2^ = 0.08). Post-test showed that the P300 amplitude was significantly greater in the PRS-conversion group than that in the HC (*p* *=* 0.001, *d* = 0.76) and PRS-complete remission groups (*p* *=* 0.016, *d* = 0.72). The P300 amplitude in the PRS-symptomatic group was significantly greater than that in the HC group (*p* *=* 0.006, *d* = 0.48). There was no significant difference in the P300 amplitude among the other groups. The main effect of stimulation type was significant (*F*(1, 173) =  13.41, *p* <  0.001, Ƞ^2^  =  0.07), and the P300 amplitudes induced by 20-Hz sound stimulation were significantly greater than those induced by 40-Hz sound stimulation. The interaction between clinical outcome group and stimulation type reached marginal significance (*F*(4, 172) = 2.18, *p* *=* 0.07, Ƞ^2^  =  0.05). Simple effect analysis showed that the P300 amplitude induced by 40-Hz sound stimulation in the PRS-conversion group was significantly greater than that in the PRS-complete remission (*p* *=* 0.003, *d* = 0.94) and HC groups (*p* *=* 0.001, *d* = 0.78). The P300 amplitude induced by 40-Hz sound stimulation was significantly greater in the PRS-symptomatic group than that in the PRS-complete remission (*p* *=* 0.011, *d* = 0.76) and HC groups (*p* *=* 0.002, *d* = 0.60). There was no significant difference in the P300 amplitude induced by 40-Hz sound stimulation among the other groups. The P300 amplitude induced by 20-Hz sound stimulation was significantly greater in the PRS-conversion group than that in the HC group (*p* *=* 0.01, *d* = 0.73). There was no significant difference in the P300 amplitude induced by 20-Hz sound stimulation among the other groups (*p* *=* 0.16–0.78, *d* = 0.03–0.18). In terms of latency, the main effect of the clinical outcome group was not significant (*F*(4, 173) = 1.48, *p* *=* 0.21, Ƞ^2^  =  0.03). The main effect of stimulation type was significant (*F*(1, 173) = 11.87, *p* = 0.001, Ƞ^2^  =  0.07), the P300 latency induced by 20-Hz sound stimulation was significantly smaller than that induced by 40-Hz sound stimulation. The interaction between clinical outcome group and stimulus frequency was not significant (*F*(4, 172) = 1.64, *p* *=* 0.17, Ƞ^2^  =  0.03). The difference waveforms and topographic maps of each clinical outcome group with different stimulus frequencies are shown in Fig. 3.

Correlation analysis showed that there was not significant correlation between the P300 amplitudes and latencies induced by 20- and 40-Hz sound stimulation in patients with PRS and their SOPS total scores, SOPS-positive symptoms scores, SOPS-negative symptoms, SOPS-disintegrating symptoms scores, and SOPS-general symptoms scores (*r* = −0.14–0.15, *p*s > 0.05). Additionally, we used multiple logistic regression to analyze the association between P300 amplitude and clinical outcomes of PRS. The specific results are presented in the Supplementary Materials.

### Reaction times and accuracies of target detection

The average reaction times (RTs) and accuracies of different groups to target stimulation are shown in Table 2. Regarding RT, the main effect of group was significant (*F*(2, 225) = 3.38, *p* = 0.036, Ƞ^2^ = 0.03). Post-hoc test showed that the average RT of PRS was significantly faster than that of ED (*p* = 0.01, *d* = 0.38), there was no statistically significant difference between PRS and HC (*p* = 0.14, *d* = 0.24), and between ED and HC (*p* = 0.40, *d* = 0.16). The main effect of stimulus frequency was significant (*F*(1, 225) = 32.16, *p*  <  0.001, Ƞ^2^  =  0.13), the average RT to 20-Hz target stimulation was significantly faster than that to 40-Hz target stimulation (*p*  <  0.001, *d* = 0.21). The interaction between the group and stimulus frequency was not significant, (*F*(2, 224) = 0.08, *p* = 0.93, Ƞ^2^ = 0.001). The main effect of clinical outcomes of PRS after follow-up was not significant (*F*(3, 120) = 0.77, *p* = 0.51, Ƞ^2^ =  0.02). The interaction between the clinical outcome group and stimulus frequency was also not significant (*F*(3, 120) = 1.32, *p* = 0.27, Ƞ^2^  =  0.03). Concerning accuracies, there were no significant differences in the accuracies among the groups (*F*(2, 225) = 1.15, *p* = 0.32, Ƞ^2^  =  0.01). Among the stimulus frequencies, the accuracy was significantly higher for 20-Hz targets than for 40-Hz targets (*F*(1, 225) = 7.17, *p* = 0.008, Ƞ^2^  =  0.031). The interaction between the group and stimulus frequency was not significant (*F*(2, 224) = 0.19, *p* = 0.83, Ƞ^2^  =  0.002). The main effect of clinical outcomes of PRS after follow-up was not significant (*F*(3, 120) = 0.73, *p* = 0.54, Ƞ^2^  =  0.02). The interaction between the clinical outcome group and stimulus frequency was also not significant (*F*(3, 120) = 1.63, *p* = 0.19, Ƞ^2^ = 0.04).

**Table 2:** Average reaction times and accuracies of different groups.

	Stimulus frequency	PRS (*n* = 122)	ED (*n* = 51)	HC (*n* = 53)	PRS-conversion (*n* = 24)	PRS-symptomatic (*n* = 56)	PRS-emotion disorder (*n* = 19)	PRS-complete remission (*n* = 22)
Reaction time (ms)	20-Hz	432.57(96.77)	476.23(120.89)	457.87(94.07)	453.97(126.68)	437.29(94.54)	407.43(56.92)	407.61(69.58)
	40-Hz	456.08(103.22)	496.26(117.06)	480.86(110.36)	469.92(119.79)	454.92(106.87)	442.65(71.44)	446.93(94.40)
Accuracy ( %)	20-Hz	99.31(0.33)	99.52(0.16)	99.96(0.01)	99.90(0.26)	99.48(0.31)	99.78(0.63)	99.76(0.99)
	40-Hz	98.73(0.46)	99.05(0.29)	99.51(0.08)	99.58(0.91)	98.63(0.44)	98.37(0.61)	98.18(0.62)

Note: Data are mean (SD). PRS = psychosis risk syndrome; ED = emotional disorder; HC = healthy control.

## Discussion

The current study used a new auditory Oddball task using rhythmic compound tone stimuli as the experimental material to evaluate the P300 responses of youth with PRS, ED, and HC individuals. We explored the specificity of the P300 responses of patients with PRS and the attention processing characteristics of participants towards rhythmic compound tone stimuli of different frequencies, and analyzed the association between the auditory P300 response induced by compound tone stimuli of different frequencies and the clinical outcomes of PRS. The results showed that compared to the ED and HC groups, the PRS group had a larger average P300 amplitude, while there was no significant difference between the ED and HC groups. These results indicate that patients with PRS exhibit abnormalities in auditory attention processing to composite sound stimuli, and P300 amplitude may be a specific electrophysiological indicator of auditory attention processing abnormalities in patients with PRS. In addition, the results showed that the P300 amplitude induced by rhythmic compound tone stimuli at 20-Hz was significantly greater than that induced by stimuli at 40-Hz, indicating that participants had different characteristics in attentional processing of rhythmic composite sound stimuli at different frequencies. Additionally, we also found that compared to individuals who completely relieved from PRS symptoms after 12 months of follow-up (PRS-complete remission) and the HC group, those who converted to psychosis (PRS-conversion) had greater P300 amplitudes. Moreover, compared to 20-Hz sound stimulation, the auditory P300 amplitude induced by 40-Hz sound stimulation tends to amplify the differences in clinical results between groups. These results indicated that the auditory P300 amplitude induced by rhythmic compound tone stimulus was associated with the clinical outcomes of PRS, and the P300 amplitude may be an important potential electrophysiological biomarker for early prediction of psychosis.

These results are analogous to but different from previous studies. Previous studies have found that compared to HCs, patients with PRS exhibit significant auditory attentional abnormalities, manifested as smaller P300 amplitudes (H. K. Hamilton et al., 2019,; Tang et al., 2020). Conversely, we found a larger P300 amplitude in patients with PRS. This difference may be due to the stimulus material, previous studies have reported that the smaller P300 amplitude in patients with PRS was induced by pure tone stimuli (Bramon et al., 2008; Tang et al., 2020), while our study used rhythmic compound tone stimuli. Compared to pure tone stimulation, processing compound sound stimulation often requires individuals to invest more cognitive resources (Blanchet, 2015; Griskova-Bulanova et al., 2018; Wickens, 1980). Research on patients with mental disorders has found that individuals with psychiatric symptoms typically exhibit slower processing of information with less demand for cognitive resources and more sensitive processing of information with more demand for cognitive resources (Aase et al., 2021; Addington et al., 2019; Vaquerizo-Serrano et al., 2022). Therefore, this study develops and enriches previous studies, demonstrating that attention processing of compound tone stimuli can reflect new features of auditory attention abnormalities in patients with PRS, revealing that patients with PRS may have two forms of auditory attentional abnormalities—attentional processing deficits to simple auditory stimuli (represented by the smaller P300 amplitude of pure tones) and attentional processing hypersensitivity to complex auditory stimuli (represented by the shorter response time of compound tone stimuli and the larger P300 amplitude).

More importantly, we found that the patients with PRS had a greater P300 amplitude induced by rhythmic compound tone stimuli than those with ED, and the symptom difference between PRS and ED was based on the existence of positive symptoms only. Therefore, the abnormal P300 amplitude in the patients with PRS may be associated with its positive symptoms, and the P300 amplitude induced by compound tone stimuli may be a specific electrophysiological indicator of PRS. In addition, we also found that participants exhibit different characteristics in their attention processing to rhythmic compound tone stimuli of different frequencies. Compared to the 20-Hz sound stimulus, participants reaction time slower and have lower accuracy to the 40-Hz target stimulus, and the P300 amplitude induced by the 40-Hz compound tone stimuli was smaller, which may be due to the higher difficulty lever of auditory discrimination during attentional processing of 40-Hz target compound tone stimuli, because for composite sound stimulation, high-frequency target sounds are more difficult to distinguish from standard sounds with the same frequency than low-frequency target sounds (Tada et al., 2016).

Furthermore, we found an association between the baseline P300 amplitude of patients with PRS and its future clinical outcomes, which is consistent with previous studies (Foss-Feig et al., 2021; H. K. Hamilton et al., 2019; Tang et al., 2020). Moreover, we have also made some new trend findings: compared to rhythmic compound tone stimuli at 20-Hz, the P300 amplitude induced by 40-Hz tone stimulation has a better discriminative effect on the future clinical outcomes of PRS. The reason may be related to the gamma neural oscillation responses caused by rhythmic sound stimulation at 40-Hz (D. Mathalon et al., 2020; Perez et al., 2013; Tada et al., 2016). Gamma neural oscillations are closely related to attentional processing (Fell et al., 2003; Parciauskaite et al., 2021) and are essential for integrating information within neural circuits (Chung, Geramita & Lewis, 2022; Shin, O'Donnell, Youn & Kwon, 2011). Previous studies have found that gamma band ASSR induced by 40-Hz sound stimulation can more effectively predict the clinical outcomes of PRS (Cortes-Briones, 2021; Grent-’t-Jong et al., 2021). These results indicate that patients with PRS who have been classified as psychosis already have significant neurological deficits related to auditory attention before reaching the diagnostic criteria (Grent-’t-Jong et al., 2021; H. K. Hamilton et al., 2019), and the rhythmic compound tone stimuli of 40-Hz may have more unique value in evaluating the neurophysiological responses related to auditory attention processing in patients with PRS.

In summary, this study validated the specificity of P300 amplitude induced by rhythmic compound tone stimuli for patients with PRS, and revealed that the participants have different characteristics in attentional processing of rhythmic compound tone stimuli of different frequencies. Moreover, the association between the auditory P300 amplitude induced by rhythmic compound tone stimuli of different frequencies and the clinical outcomes of PRS was analyzed, and the key neuroelectrophysiological indicator related to the clinical outcomes of PRS were identified. This study supplements the shortcomings of previous studies and develops and enriches the neurophysiological mechanisms of auditory attention abnormalities in PRS, which is of great significance for a deeper understanding of the neurophysiological characteristics and clinical transformation characteristics of PRS. Furthermore, the findings of this study indicated that the P300 amplitude induced by rhythmic compound tone stimuli at 40-Hz has the potential as an electrophysiological biomarker for individualized psychiatric risk assessment in patients with PRS. By adding objective clinical information, the accuracy and reliability of individual risk assessment in PRS can be improved. In addition, the P300 amplitude induced by 40-Hz rhythmic compound tone stimuli may play a more effective role in developing clinical staging algorithms, laying the foundation for early active intervention, targeted treatment, and prognosis evaluation for high-risk individuals with psychosis.

However, this study has some limitations. For example, the follow-up time of youth with PRS is only 1 year, which may limit the investigating on the relationship between early auditory attention processing and the onset time of psychosis. Besides, to better match sound stimuli in this study, we only used rhythmic compound tone stimuli as experimental stimuli, without simultaneously using pure tone stimuli. This makes it impossible for us to analyze the auditory attention processing characteristics of participants on both pure tone and rhythmic composite tone stimuli in the same sample. In addition, the relationship between the severity of individual symptoms in PRS and their neurophysiological responses is very complex (Feczko et al., 2019), and linear correlation may not be sufficient to reveal this complex relationship, this study did not conduct a more in-depth analysis on this. In future studies, the follow-up time of patients with PRS should be increased to fully discover the relationship between early auditory attention processing and the onset time of psychosis in high-risk individuals. Additionally, the assessment task with pure tone as stimulus material should be added to the existing assessment task, to comprehensively analyze the auditory attentional processing characteristics of pure tone and rhythmic composite tone stimuli. Moreover, future studies can use more advanced analytical techniques to conduct in-depth analysis of the relationship between the severity of PRS individual symptoms and their auditory P300 response, to clarify the relationship between symptom severity and neurophysiological responses.

## Conclusion

In conclusion, our study extends previous findings and reveals that the auditory P300 amplitude induced by rhythmic compound tone stimuli is a specific electrophysiological manifestation of PRS, and the P300 amplitude induced by 40-Hz stimulation may be sensitive to the clinical outcomes of PRS, including both conversions to psychosis and clinical complete remission.

## Funding

This work was supported by 10.13039/501100001809National Natural Science Foundation of China (grant number 32171040 & 32371105, to Dr. Antao Chen), the Sichuan Provincial Health Commission of Science and Technology Project (grant number 20PJ281, to Dr. Yongqing Hou), and the Key R&D Projects of Science and Technology Plan of Science & Technology Department of Sichuan Provincial (grant number 2023YFS0085, to Dr. Yongqing Hou). These funds had no role in the design and conduct of the study; collection, management, analysis, and interpretation of the data; preparation, review, or approval of the manuscript; and decision to submit the manuscript for publication. The authors report no financial relationships with commercial interests.

## Data availability

The data that support the findings of this study are available from the corresponding author, A.C., upon reasonable request.

## Declaration of competing interest

The authors declare that there are no conflict of interests.

## Appendix Supplementary materials

application 1 Image, application 1
