# Supplementary material for: The specificity of the auditory P300 responses and its association with clinical outcomes in youth with psychosis risk syndrome
Source: Int J Clin Health Psychol. 2024 Jan 11;24(1):100437. doi: 10.1016/j.ijchp.2024.100437 (PMC10825643; doi:10.1016/j.ijchp.2024.100437)
Supplement: application 1 [file mmc1.docx]

**The specificity of the auditory P300 responses and its association with clinical outcomes in youth with psychosis risk syndrome**

**Supplementary Materials**

*Specific diagnostic information for participants*

Psychiatrists with over two years of clinical psychological work experience and rigorous evaluation training will used the Chinese version of the Structured Interview for Psychosis-Risk Syndromes (SIPS) and scale of psychosis-risk symptoms (SOPS) (Zheng et al., 2012) to evaluate and screen patients with PRS who meet the requirements of this study. Patients with PRS were assessed to meet one of the three types of PRS and included in the PRS group. The three types of PRS are Brief Intermittent Psychotic State (BIPS), Attenuated Positive Symptom State (APSS) and Genetic Risk and Deterioration State (GRDS) (Miller et al., 2003; Yung et al., 2006). BIPS is a recent and very brief positive symptom of psychosis (including unusual thinking content, delusional mood, implicated beliefs, perceptual abnormalities, and speech disorders) that typically occurs within the past 3 months and reaches a severe level of psychosis. APSS is a threshold positive symptom that has occurred within the past year and reached sufficient severity and frequency. GRDS is a genetic risk associated with schizophrenia spectrum disorders, and its functional deterioration in the past year has exceeded 30%. Among the 122 individuals who met the PRS criteria in this study, 1 was BIPS, 116 were APSS, 3 were GRDS, and 2 were both GRDS and APSS compliant. In addition, this study used the Mini-International Neuropsychiatric Interview (MINI) (Si et al., 2009) to exclude other mental disorders.

For patients with emotional disorders (ED), the Patient Health Questionnaire (PHQ-9) ) (Kroenke et al., 2001) and Generalized Anxiety Disorder-7 (GAD-7) (Spitzer et al., 2006) questionnaire were used to conduct preliminary evaluations on participants, and then self-assessment was conducted on participants who met the initial evaluation criteria using Self-Rating Depression Scale (SDS) (Zung, 1965) and Self-Rating Anxiety Scale (SAS) (Zung, 1971). At the same time, a psychological evaluator with two years of clinical psychological work experience used the Hamilton Depression Scale (HAMD) (Williams & Janet, 1988) and Hamilton Anxiety Scale (HAMA) (Snaith et al., 1982) to assess the emotional status of participants. Additionally, psychiatrists used SIPS and MINI to diagnose and evaluate the participants. Individuals with emotional disorders who are enrolled must meet the diagnosis of the current depressive episode and have symptoms of generalized anxiety disorder. Based on the results of initial evaluation, self-evaluation, and other evaluations, individuals with emotional disorders must meet the criteria of PHQ-9 total score ≥ 10 points, HAMD-17 total score ≥ 17 points, SDS standard score ≥ 63 points, and/or GAD-7 total score ≥ 10 points, HAMA total score ≥ 14 points, and SAS standard score ≥ 60 points. In addition, individuals with emotional disorders need to meet the criteria of not meeting any type of PRS assessed by SIPS and having no other mental disorders assessed by MINI.

Healthy control individuals (HC) were evaluated by SIPS to exclude PRS, and assessed by MINI to exclude all other mental disorders. Moreover, HC must meet the criteria of the PHQ-9 total score < 3 points, HAMD-17 total score < 6 points, SDS standard score < 50 points, and GAD-7 total score < 3 points, HAMA total score < 6 points, and SAS standard score < 50 points. In addition, HC participants who had previously suffered from mental illness or whose first-degree relatives had mental illness were also excluded.

All participants followed the following exclusion criteria: 1) had nervous system diseases; 2) had a history of traumatic brain injury; 3) had taken psychotropic drugs in the past year; 4) had received electroconvulsive therapy; 5) were pregnant or lactating; 6) had a history of alcohol abuse or dependence; or 7) had a history of using or relying on heroin, morphine, or similar substances.

*The Association Between P3 Amplitudes and Clinical Outcomes of PRS*

Based on the P300 amplitude induced by 20- and 40-Hz sound stimulation, the clinical outcomes of PRS were predicted. Multiple logistic regression analysis showed that the model passed the fitting test (χ*^2^* = 15.14, *p =* 0.019). Using the PRS-complete remission group as the reference, the P300 amplitude induced by 40-Hz sound stimulation had a significant effect in the PRS-conversion (*B* = 0.45, Wald = 8.93, *p =* 0.003; Exp(B) = 1.56; 95% CI: 1.17–2.10) and PRS-symptomatic groups (*B* = 0.40, Wald = 8.94, *p =* 0.003; Exp(B) = 1.49; 95% CI: 1.15–1.93), but not in the PRS-emotional disorder group (*B* = 0.21, Wald = 1.88, *p =* 0.17); this indicates that compared with the PRS-complete remission group, a greater P300 amplitude induced by 40-Hz sound stimulation was associated with a higher probability of categorizing individuals with PRS into the PRS-conversion and PRS-symptomatic groups. When considering the PRS-conversion group as the reference, the P300 amplitude induced by 40-Hz sound stimulation had a significant effect in the PRS-complete remission group (*B* = -0.45, Wald = 8.93, *p =* 0.003; Exp(B) = 0.64; 95% CI: 0.48–0.86), but not in the PRS-symptomatic (*B* = -0.05, Wald = 0.23, *p =* 0.63) and PRS-emotional disorder groups (*B* = -0.24, Wald = 2.82, *p =* 0.09); this indicates that compared with the PRS-conversion group, a greater P300 amplitude induced by 40-Hz sound stimulation was associated with a lower probability of categorizing individuals with PRS into the PRS-complete remission group. In addition, when considering the PRS-symptomatic group as the reference, the P300 amplitude induced by 40-Hz sound stimulation had a significant effect in the PRS-complete remission group (*B* = -0.40, Wald = 8.94, *p =* 0.003; Exp(B) = 0.67; 95% CI: 0.52–0.87), but not in the PRS-conversion (*B* = 0.05, Wald = 0.23, *p =* 0.63) and PRS-emotional disorder groups (*B* = -0.19, Wald = 2.24, *p =* 0.14); this indicates that compared with the PRS-symptomatic group, a greater P300 amplitude induced by 40-Hz sound stimulation was associated with a lower probability of categorizing individuals with PRS into the PRS-complete remission group. The P300 amplitude induced by 20-Hz sound stimulation had no significant effect on the clinical outcome of each group.

**Table A.1**

Demographic characteristics of clinical outcome groups of PRS after follow-up.

| **Characteristic** | **PRS-conversion (*n*=24)** | **PRS-symptomatic (*n*=56)** | **PRS-emotion disorder (*n*=19)** | **PRS-complete remission (*n*=22)** | ***p*-Value** |
| --- | --- | --- | --- | --- | --- |
| Age in years, mean (*SD*) | 18.21(1.06) | 18.52(0.87) | 18.68(0.89) | 18.41(1.26) | 0.43 |
| Sex assigned at birth, no. (%) |  |  |  |  | 0.68 |
| Male | 9(37.5%) | 20(35.7%) | 7(36.8%) | 5(22.7%) |  |
| Female | 15(62.5%) | 36(64.3%) | 12(63.2%) | 17(77.3%) |  |
| Home location, no. (%) |  |  |  |  | 0.41 |
| City | 10(41.7%) | 25(44.6%) | 7(36.8%) | 5(22.7%) |  |
| Urban rural fringe | 5(20.8%) | 8(14.3%) | 2(10.5%) | 7(31.8%) |  |
| Countryside | 9(37.5%) | 23(41.1%) | 10(52.6%) | 10(45.5%) |  |
| Father's education, no. (%) |  |  |  |  | 0.58 |
| Junior high school and below | 12(50.0%) | 29(51.8%) | 12(63.2%) | 12(54.5%) |  |
| High school and technical secondary school | 4(16.7%) | 15 (26.8%) | 2(10.5%) | 8(36.4%) |  |
| Junior college | 5(20.8%) | 5(8.9%) | 2(10.5%) | 2(9.1%) |  |
| Undergraduate | 3(12.5%) | 6(10.7%) | 3(15.8%) | 0(0.0%) |  |
| Graduate and above | 0(0.0%) | 1(1.8%) | 0(0.0%) | 0(0.0%) |  |
| Mother's education, no. (%) |  |  |  |  | 0.81 |
| Junior high school and below | 11(45.8%) | 33(58.9%) | 13(68.4%) | 14(63.6%) |  |
| High school and technical secondary school | 8(33.3%) | 15(26.8%) | 3(15.8%) | 6(27.3%) |  |
| Junior college | 3(12.5%) | 6(10.7%) | 1(5.3%) | 1(4.5%) |  |
| Undergraduate | 2(8.3%) | 1(1.8%) | 2(10.5%) | 1(4.5%) |  |
| Graduate and above | 0(0.0%) | 1(1.8%) | 0(0.0%) | 0(0.0%) |  |

Note: Data are number (percentage) or mean (SD), when appropriate. P-values by ANOVA linear term or Pearson Chi square tests (for linear association). PRS = psychosis risk syndrome.

**Table A.2**

Statistics on anxiety, depression, and SOPS scores of individuals with PRS, ED, and HC.

| **Variables** | **PRS (*n*=122)** | **ED (*n*=51)** | **HC (*n*=53)** |
| --- | --- | --- | --- |
| GAD-7 score | 8.82(5.31) | 9.48(4.79) | 0.13(0.62) |
| PHQ-9 score | 10.70(5.63) | 11.06(5.15) | 0.21(0.95) |
| SAS standard score | 47.57(8.99) | 46.44(8.36) | 32.24(3.91) |
| SDS standard score | 55.40(11.39) | 55.63(8.69) | 34.43(5.72) |
| HAMA score | 11.41(6.59) | 18.65(7.03) | 0.81(1.23) |
| HAMD score | 12.14(5.69) | 18.29(6.39) | 0.83(1.16) |
| SOPS total score | 19.44(14.01) | 21.00(8.97) | 0.25(0.59) |
| SOPS-positive symptoms | 7.38(4.01) | 0.90(0.91) | 0.04(0.27) |
| SOPS-negative symptoms | 5.71(4.94) | 8.58(5.32) | 0.08(0.27) |
| SOPS-disintegrating symptoms | 1.98(2.88) | 3.62(1.74) | 0.08(0.27) |
| SOPS-general symptoms | 4.37(4.47) | 7.90(3.55) | 0.06(0.23) |

Note: Data are mean (SD). PRS = psychosis risk syndrome; ED = emotional disorder; HC = healthy control; GAD-7 = Generalized Anxiety Disorder-7 questionnaire; PHQ-9 = Patient Health Questionnaire; SAS = Self-Rating Anxiety Scale; SDS = Self-Rating Depression Scale; HAMA = Hamilton Anxiety Scale; HAMD = Hamilton Depression Scale; SOPS = Scale of Psychosis-risk Symptoms.


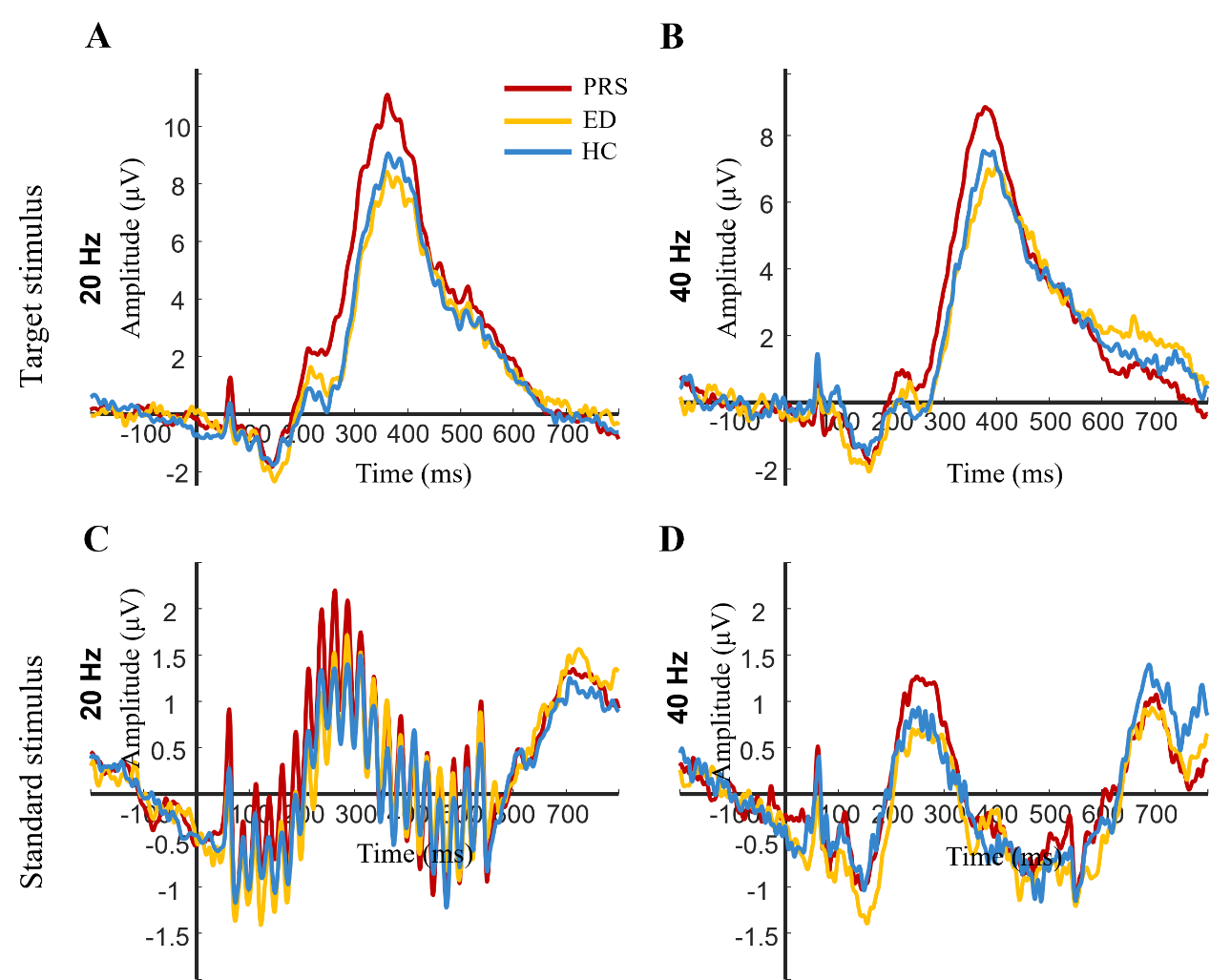


**Fig. A.1.** The raw pre-subtraction waveforms induced by target and standard stimuli of PRS, ED, and HC. (A) The raw waveforms induced by the 20-Hz target stimulus of the three groups. (B) The raw waveforms induced by the 40-Hz target stimulus of the three groups. (C) The raw waveforms induced by the 20-Hz standard stimulus of the three groups. (D) The raw waveforms induced by the 40-Hz standard stimulus of the three groups. All waveforms are the average of P1, PZ, P2, and POZ electrodes. PRS = psychosis risk syndrome; ED = emotional disorder; HC = healthy control.


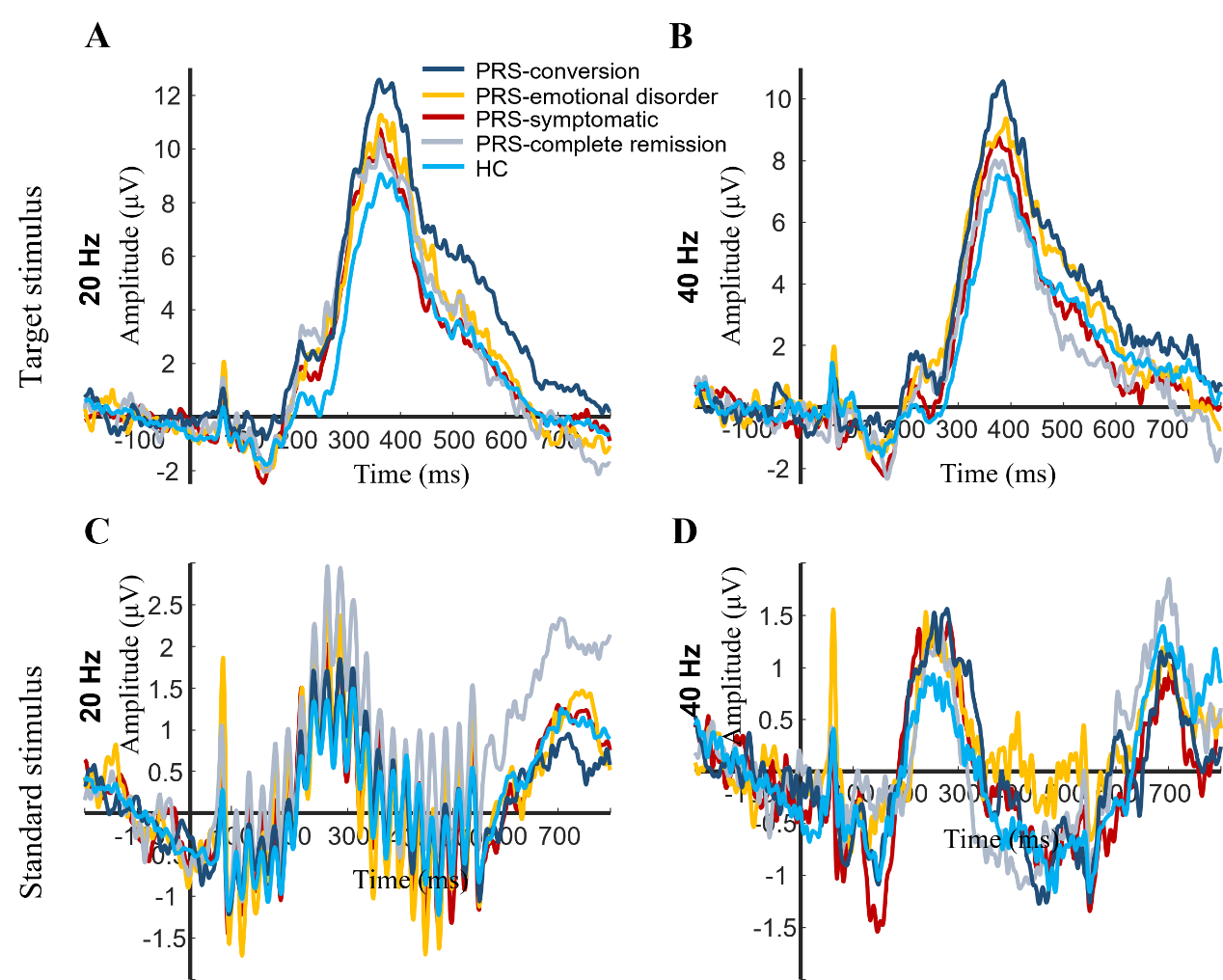


**Fig. A.2.** The raw pre-subtraction waveforms induced by target and standard stimuli of PRS clinical outcome groups and HC group**.** (A) The raw waveforms induced by the 20-Hz target stimulus of PRS clinical outcome groups and HC group. (B)The raw waveforms induced by the 40-Hz target stimulus of PRS clinical outcome groups and HC group. (C) The raw waveforms induced by the 20-Hz standard stimulus of PRS clinical outcome groups and HC group. (D) The raw waveforms induced by the 40-Hz standard stimulus of PRS clinical outcome groups and HC group. All waveforms are the average of P1, PZ, P2, and POZ electrodes. PRS = psychosis risk syndrome; HC = healthy control.

**References**

Kroenke, K., Spitzer, R. L., & Williams, J. B. W. (2001). The PHQ-9: Validity of a brief depression severity measure. *Journal of General Internal Medicine*, *16*(9), 606–613. https://doi.org/10.1046/j.1525-1497.2001.016009606.x

Miller, T. J., McGlashan, T. H., Rosen, J. L., Cadenhead, K., Ventura, J., McFarlane, W., Perkins, D. O., Pearlson, G. D., & Woods, S. W. (2003). Prodromal Assessment with the Structured Interview for Prodromal Syndromes and the Scale of Prodromal Symptoms: Predictive Validity, Interrater Reliability, and Training to Reliability. *Schizophrenia Bulletin*, *29*(4), 703–715. https://doi.org/10.1093/oxfordjournals.schbul.a007040

Si, T., Su, L., Dang, W., Su, Y., Chen, J., Dong, W., Kong, Q., & Zhang, W. (2009). Evaluation of the reliability and validity of Chinese version of the Mini-International Neuropsychiatric Interview in patients with mental disorders. *Chinese Mental Health Journal*, *23*(7), 493–497. https://doi.org/10.3969/j.issn.1000-6729.2009.07.011

Snaith, R. P., Baugh, S. J., Clayden, A. D., Husain, A., & Sipple, M. A. (1982). The Clinical Anxiety Scale: An instrument derived from the Hamilton Anxiety Scale. *Br J Psychiatry*, *141*(5), 518–523. https://doi.org/10.1192/bjp.141.5.518

Spitzer, R. L., Kroenke, K., Williams, J. B. W., & Löwe, B. (2006). A Brief Measure for Assessing Generalized Anxiety Disorder: The GAD-7. *Archives of Internal Medicine*, *166*(10), 1092–1097. https://doi.org/10.1001/archinte.166.10.1092

Williams, & Janet, B. W. (1988). A structured interview guide for the Hamilton Depression Rating Scale. *Archives of General Psychiatry*, *45*(8), 742–747. https://doi.org/10.1037/t67131-000

Yung, A. R., Stanford, C., Cosgrave, E., Killackey, E., Phillips, L., Nelson, B., & McGorry, P. D. (2006). Testing the Ultra High Risk (prodromal) criteria for the prediction of psychosis in a clinical sample of young people. *Schizophrenia Research*, *84*(1), 57–66. https://doi.org/10.1016/j.schres.2006.03.014

Zheng, L., Wang, J., Zhang, T., Li, H., Li, C., & Jiang, K. (2012). Reliability and validity of the Chinese version of Scale of Psychosis-risk Symptoms. *Chinese Mental Health Journal*, *26*(8), 571–576. https://doi.org/10.3969/j.issn.1000-6729.2012.08.003

Zung, William. W. K. (1965). A Self-Rating Depression Scale. *Archives of General Psychiatry*, *12*, 63–70. https://doi.org/10.1001/archpsyc.1965.01720310065008

Zung, William. W. K. (1971). A Rating Instrument for Anxiety Disorders. *Psychosomatics*, *12*(6), 371–379. https://doi.org/10.1016/S0033-3182(71)71479-0
